# Only empathy-related traits, not being mimicked or endorphin release, influence social closeness and prosocial behavior

**DOI:** 10.1038/s41598-023-30946-9

**Published:** 2023-03-11

**Authors:** Birgit Rauchbauer, Gabriela Jank, Robin I. M. Dunbar, Claus Lamm

**Affiliations:** 1 Social, Cognitive and Affective Neuroscience Unit, Department of Cognition, Emotion, and Methods in Psychology, Faculty of Psychology, University of Vienna, Liebiggasse 5, 1010 Vienna, Austria; 2 Vienna Cognitive Science Hub, University of Vienna, Universitätsstraße 7, 1010 Vienna, Austria; 3 Laboratoire Parole et Langage, Aix-Marseille Université, CNRS, 5 Avenue Pasteur, 13100 Aix-en-Provence, France; 4 Laboratoire de Neurosciences Cognitives, Aix-Marseille Université, CNRS, 3, Place Victor-Hugo, 13331 Marseille Cedex 3, France; 5 Social and Evolutionary Neuroscience Research Group, Department of Experimental Psychology, University of Oxford, Anna Watts Building, Radcliffe Observatory Quarter, Woodstock Rd, Oxford, OX26GG UK; 6 Institute of Language, Communication and the Brain, 13100 Aix-en-Provence, France

**Keywords:** Psychology, Endocrinology

## Abstract

Seminal studies suggest that being mimicked increases experienced social closeness and prosocial behavior to a mimicking confederate (i.e., interaction partner). Here we reexamine these results by considering the role of empathy-related traits, an indirect proxy for endorphin uptake, and their combined effects as an explanation for these results. 180 female participants were mimicked or anti-mimicked in an interaction with a confederate. The effects of being mimicked versus anti-mimicked in relation to empathy-related traits and endorphin release (assessed indirectly via pain tolerance) on experienced closeness and prosocial behavior were assessed using Bayesian analyses. Our results suggest that high individual empathy-related traits increase social closeness to the anti-mimicking and mimicking confederate and to one’s romantic partner, as compared to mimicry alone. Results furthermore strongly suggest that high individual empathy-related traits increase prosocial behavior (donations and willingness to help) as compared to mimicry alone. These findings extend previous work by highlighting that empathy-related traits are more influential in creating positive effects on social closeness and prosocial behavior than a one-shot mimicking encounter.

## Introduction

Whether during a coffee break at work, or engaging with friends, family, and new acquaintances, we unconsciously align our postures to our interaction partner—we mimic each other. Being mimicked increases social closeness and prosocial behavior (social closeness e.g.,^1,2^; prosocial behavior e.g.,^3,4^). Yet, the influence of mimicry on social closeness and prosocial behavior may be modulated by a myriad of individual trait and bodily variables.

Countless personality trait and psychophysiological factors interact to shape our interactive behavior. On the side of personality traits, empathy-related traits have been ascribed a prominent influence on social closeness (e.g.,^5–7^) and prosocial behavior (e.g.,^5,8–11^). Among the psychophysiological factors, various neurotransmitters, such as dopamine, serotonin, endorphin, oxytocin or vasopressin, have been ascribed social functions (e.g.,^12^). Amid those, only the endorphin system’s link to social closeness and prosocial behavior has been studied in relation to synchronous behavior (for effects on social closeness see e.g.,^13–15^; for prosocial behavior see e.g.,^16^). Synchronous behavior differs from mimicry only in the temporal dimension of interactional motor alignment (synchronous behaviour is temporally overlapping, whereas mimicry has a temporal delay of 3–5 s^1^).

In this article we re-examine the effects of being mimicked on social closeness and prosocial behavior in relation to empathy-related traits and an indirect measure of endorphin release. We will compare being mimicked with topographically isomorph movements to the control condition - being anti-mimicked with heteromorph (i.e., moving different body parts) but temporally contingent (i.e., same temporal delay as when being mimicked) movements. This is based on previous results from our group in which social closeness and prosocial behaviour relied on topographically isomorphic movements^17^. It is still debated whether topographically isomorphic^17^, perceived contingency (i.e., predictive relationship)^18^, or high contiguity (i.e., temporal proximity) of movements^19^ and/or effector matching^20^ affect social behavior. This study extends previous experiments in which being mimicked was compared to a control condition with no movement from the confederate. To keep the amount of movement constant across conditions, we chose to compare two potential forms of being mimicked, both contingent and contiguous on the participant’s behavior, but with different degrees of isomorphism.

Mimicry’s positive social effects are well-documented throughout development (rev.^21^). While neonates’ abilities for interpersonal motor alignment has prominently been claimed^22^ and debated (e.g.,^23,24^), social contingencies between the caregiver and infants in their first months of life may build the foundation for interactions based on motor and rhythmic alignment^25^. As a “social glue”, mimicry may evolutionarily be designed for the establishment and maintenance of social relations^2^. Infants around one year of age already infer third-party affiliations from synchronous interaction^26,27^. By this stage, they imitate their peers as a way of increasing group cohesion^28^. Mimicry may also increase social closeness and prosocial behaviour towards the mimicking interaction partner in adults^4,29,30^. Its positive effects on prosocial behavior extend to an unknown experimenter, charities^3,4,30^, and strangers^31^. Recently we found no relation of being mimicked and empathy-related traits (perspective taking) on social closeness to the confederate (i.e., interaction partner) measured via a rating scale^32^. In this article, we use interpersonal space^33^ as a measure of closeness. Interpersonal space between oneself and another changes as a function of experienced relational closeness, and gets smaller the closer the relationship^34^, serving as a reliable indicator of affiliative relationship^35^. While measuring interpersonal distance is artificial if provided on a computer screen, it represents an easily implementable way of measuring social closeness. Interpersonal distance has been shown to be influenced by oxytocin administration and empathy^7^. We aim to test the joint influence of mimicry, empathy-related traits and endorphin release on social closeness and prosocial behavior.

Specifically, two facets of empathy-related traits, namely empathic concern and perspective taking, seem important for social, but especially prosocial, behavior. Empathic concern (e.g.,^8,36^; also compassion) is an inherently other-oriented emotional response, “feeling *for* somebody”^8^, rather than “feeling *as* the other”, which is dominant in affect sharing^10^. It motivates amelioration of the other’s state^9^, without self-focus or the wish to alleviate personal distress^36^. Perspective taking requires the activation of one’s own cognitive representation of another’s affective state. (Empathic) perspective taking includes access to, and understanding of, another’s personal representations of specific needs or situations, while being aware that it is different and separate from one’s own^9^. It also facilitates prosocial behavior once it has been understood that the other needs help or assistance^37^. The link between prosocial behavior and empathy-related traits may be established in a sequence of affect-sharing leading to understanding of the interaction partner’s feelings, other-related concern, and finally, the engagement in prosocial behavior^38^. In this study we aimed to investigate how participants judged their willingness to help the mimicking or anti-mimicking interaction partner and their general willingness to donate to a charity.

Empathic abilities and dyadic relationship quality have been associated with variation in the β-endorphin receptor gene, OPMR1^12^. Opioids seem to play an important role in the evolution of primate sociality and social bonding (e.g.,^39,40^). Β-endorphin, the most potent endogenous opioid peptide with a high affinity for the μ-opioid receptor, is primarily implicated in regulating both physical and emotional pain and stress^41,42^. Its release causes a mild analgesia through a light opiate “high”, which also increases well-being. It is involved in hedonistic reward, including the rewarding effects of social interaction^43,44^. This has been confirmed by studies showing that the administration of the μ-opioid receptor antagonist naltrexone induces feelings of reduced social connection^45,46^. Other neurotransmitters, such as dopamine, serotonin, oxytocin or vasopressin, are also involved in regulating social behavior (e.g.,^12^) and some also have analgesic effects (e.g.,^47,48^). They may work in concert with the opioid system^40^. Synchronous activity may represent an equivalent of grooming at a distance^49^, activating increasing β—endorphin release in monkeys^50^. Endorphin increase has been shown using Positron Emission Topography (PET) scanning in humans engaging in synchronous social laughter^51^. Because endorphins do not cross the blood–brain-barrier^52^, central endorphin release is difficult to measure directly; instead, many studies have used the proxy of an increase in pain threshold and pain tolerance as a measure, thus using an indirect measure of endorphin release (e.g.,^13,15,53–55^). Taking advantage of the easy implementation of pain tolerance measures as compared to blood measures or psychopharmacologic interventions, we also used pain tolerance as a proxy for indirect endorphin release (see below for limitations of this measure). Naltrexone intervention studies confirm that behaviours that elevate pain thresholds (such as the wall-sit task used in the present study) do so via their effect on the endorphin system (e.g.,^56,57^).

Thus, overall, we expected best predictive power for models of social closeness and prosocial behavior including being mimicked (versus anti-mimicked), empathy-related traits, endorphin release (via increase in pain tolerance) and their interaction, as compared to only being mimicked (versus anti-mimicked). Specifically, we expected that being mimicked leads to an increase in social closeness to the mimicking interaction partner (i.e., confederate) in individuals with high scores on empathy-related traits and increased endorphin release (pain tolerance) as compared to only being mimicked. We also hypothesized that, if the positive effects of being mimicked in interaction with the covariates (perspective taking, empathic concern, endorphin release) generalizes to other strangers, this model would better predict increased social closeness to the experimenter than only being mimicked. In line with this, if these effects generalize, we expect the model of being mimicked in interaction with high scores on empathy-related traits and an increase in endorphin release to better predict social closeness to significant others (loved ones: mother, romantic partner, best friend) than only being mimicked. Yet, this has been shown not to occur in the context of synchronized dancing^58^. Thus, this remains an open question. Furthermore, we would expect best predictive power for the model of being mimicked (versus anti-mimicked), the covariates of empathy-related traits, endorphin release (increased pain tolerance) and their interaction on prosocial behavior as compared only being mimicked. Specifically, we expect increased individual donations to charity and willingness to help the mimicking confederate in mimicked participants scoring high on empathy-related traits with an increase in endorphin release as compared to only being mimicked.

## Methods

### Participants

180 female participants (the same participants as in Rauchbauer et al.^32^) took part in the study (mean age = 23.41 years; SD = 4.57). We estimated sample size a priori on the results of a pilot study investigating change in endorphin release via Mimicry or Anti-Mimicry condition (G*Power^59^; see supplementary material).

Female participants between 18 and 40 were included in the study. Inclusion criteria were right-handedness, no history of psychiatric or neurologic disorders, no intake of psychopharmacologic medication. Exclusion criteria were the consummation of painkillers or alcohol on the day of the experiment, current knee, meniscus or cruciate ligament problems or recent knee surgery (< 2 months). Ethical approval was granted by the ethics committee of the University of Vienna. The study was performed in accordance with the Declaration of Helsinki (and revision, 2013).

### Procedure

Participants were invited to an experiment ostensibly investigating the link between pain and visual perception in a group setting. They were paired with another female participant, who, in reality, was one of, in total, three confederates (three different female confederates in total in the project, one in the pilot, two in the main study).

Participants were randomly assigned to one of the two Mimicry groups (between-subject factor: being mimicked versus being anti-mimicked). It was ensured that the participant and the confederate did not know each other. Participants signed an informed consent form and received information about the procedure of the experiment upon entering the lab. They received 10 Euros as financial compensation at the end of the experiment.

The confederate and the participant were accompanied into different rooms to measure their individual pain threshold before being mimicked or anti-mimicked (baseline pain threshold, Timepoint 1 (T1)). We used a standard wall sit ski exercise (e.g.,^13^; details below). After this, the confederate and the participant completed the ostensible “visual perception task” together, which was the Mimicry induction: being mimicked versus anti-mimicked (details below). This was followed by the second pain threshold measure (change measure measuring pain tolerance; Timepoint 2 (T2)). Several computerized tasks were then presented to the participant, investigating the social cognitive dependent variables (published in^32,60^). The present article focusses on social closeness and individual prosocial behavior.

Social closeness, measured via a proxy of interpersonal closeness (i.e., the inverse of interpersonal distance in percent) assessed using the Comfortable Interpersonal Distance Measure (as in^61^). General prosocial behavior was assessed with actual donations to a charity. Individual willingness to help the mimicking interaction partner (i.e., the confederate) was assessed using a rating scale. Participant’s empathy-related traits were measured using the German version of the Interpersonal Reactivity Index^62^. The computerized tasks took 20–25 min in total. In a manipulation check, participants were asked about their general impressions of the experiment and whether they wanted to mention anything they found noteworthy. Noticing the mimicry induction would have led to exclusion of the data.

### Experimental set-up and stimuli

#### Mimicry versus anti-mimicry

In a between-subject design, confederates either mimicked or anti-mimicked participants’ postures (i.e., participants were either mimicked or anti-mimicked) during an interaction in which they took turns describing pictures (after^1^). Confederates mimicked participants’ posture changes in a topographically isomorph manner^1^. Confederates anti-mimicked participants’ postures by topographically misaligning their postures to the participant’s (also^63,64^). Timing of topographic alignment or misalignment was presented by the confederate with a delay of 3–5 s after participants’ posture change^1^. We chose anti-mimicry in contrast to an inactive or passive control condition where no movement was shown by the confederate, to keep motor activity level constant across conditions. This allowed us to investigate the positive effects of topographically isomorph versus heteromorph postures, controlling for changes in activity levels. This means, when mimicking, confederates would always mirror the participants’ behavior, e.g., if participant leans forward, they would also lean forward; when anti-mimicking, if the participant would lean forward, the confederate would, for example, cross her legs.

Participants and confederates were seated facing each other in a 45° angle at the same table. Each of them had a set of ten images taken from the International Affective Picture System (IAPS^65^; see Table S1) in front of them. They were asked to take turns describing the picture in front of them for one min. Pictures were completely randomized before the start of the experiment. The participant always started to describe the picture. The experimenter timed the description with a stopwatch and stopped it after a minute. The participants were asked to sort the picture to the back of their pile and the confederates took their turn in describing the picture in front of them. The first run (the “trial run”, ostensibly for task familiarization) consisted of six pictures in total (three each for participant and confederate; an interaction of 6 min) and was followed by a five-minute short break during which the participant and the confederate were separated. The second run (the “actual task”) consisted of fourteen pictures in total (seven each participant and confederate; an interaction of fourteen minutes). The tasks were split up with a break to allow enough time for participants to recover from the baseline pain threshold measure. This was done so that a potential decline in pain threshold at timepoint 2 of the wall sit ski exercise (see below) was not due to prior exhaustion. Total interaction time was twenty minutes. Participants consented to have the interaction videotaped. Confederates were instructed to deliver natural responses, including pauses and filled breaks, showing natural, polite, behavior refraining from smiles, eye contact, laughter, or touch.

#### Measure of pain tolerance as proxy for endorphin release

We used a standard wall sit ski exercise to measure pain tolerance^13,66,67^. For this, participants lean against a wall with their knees at a 90° angle and hold this position until they cannot hold it any longer. This task becomes painful quickly. The wall-sit ski exercise is less susceptible to subjective bias than other indirect measures of endorphin release, as e.g., the pressure cuff test in which pain tolerance is measured using inflation of a blood pressure cuff^66^. The amount of time in the position is measured in milliseconds. Change in pain tolerance from before to after being mimicked or anti-mimicked was used as a proxy of indirect endorphin release^66^. The experimenter gave instructions according to a script, and showed polite, neutral behavior, refraining from de-/motivating remarks.

#### Social closeness: comfortable interpersonal distance measure

We administered a digital version of the Comfortable Interpersonal Distance measure (CID) (as in^61^) using E-Prime 2.0 software^68^ to measure social closeness (in^61^ as social distance measure, which is the inverse of the closeness measure).

Participants were asked to imagine themselves as a stickfigure in the middle of the circle, while another stickfigure was approaching them from the edge. The person from the edge of the circle approached the participant and was to be stopped with a button click at a comfortable distance (colliding in case it was not stopped). The task consisted of a trial for each of the five approaching individuals of diverse relationship qualities and strengths: the (anti-) mimicking interaction partner (i.e., confederate), the experimenter, the best friend, one’s mother and romantic partner. Three practice trials of famous people were presented. The animation consisted of fifty frames in total, with the outer edge of the circle at 0 frames and the middle of the circle with the participant stickfigure at 50 frames. Frames transitioned every 90 ms, simulating the approach from the edge (frame = 0) to the middle of the circle (participant; frame = 50). We measured the closeness of the participant to the other person in percentage of frames from 0% closeness at the edge, to 100% in the middle of the circle.

#### Prosocial behavior: individual donations and willingness to help the other participant

At the end of the experiment participants were paid ten euros in coins of 50 cents. The experimenter informed participants of a collection for “Doctors without borders”. Participants could contribute on their way out if they wished to. The experimenter left the room, giving the participant unobserved space to donate or not. The final sum of 146.14 Euros was donated at the end of the project. The willingness to help the mimicking or anti-mimicking interaction partner was investigated using a 12-point scale from “not at all” to “very much” to the question “How willing are you to help the interaction partner?”. Other ratings were conducted, including on the trustworthiness and likeability of the confederate and how much sympathy they felt towards her (see supplementary material, S3.2).

#### Measure of empathy-related traits: Interpersonal Reactivity Index (IRI)

We used the German version of the Interpersonal Reactivity Index^69^ (IRI; German version^62^). Empathy-related traits in the IRI are measured on the scales of perspective taking, empathic concern, fantasy, and personal distress. The IRI overall consists of 28 items that are rated on a 5-point Likert scale, ranging from 0 (“does not describe me very well”) to 4 (“describes me very well”). Each scale is measured on 6 questions. The average score of these questions is taken as the value for each respective scale. The Cronbach’s α rating were .70 for perspective taking, .71 for empathic concern, .80 for fantasy and .60 for personal distress.

### Data analysis

We applied outlier correction using boxplots and excluded values deviating more than 1.5 times the interquartile range from the median. Bayesian ANCOVAs were conducted using JASP (https://jasp-stats.org/;^70^). Bayes factors are interpreted after the classification scheme of Wagenmakers et al., (2018)^71^.

We evaluated the predictive performance of the Bayesian ANCOVA models for the dependent variables of social closeness and prosocial behavior including the between-subject factor Mimicry Group (being mimicked versus anti-mimicked), empathy-related traits (individual perspective taking and empathic concern scores) and endorphin release (via the proxy of pain threshold) in comparison to the model including only Mimicry Group (i.e., being mimicked versus anti-mimicked). This yielded five Bayesian ANCOVA models for the five dependent variables of social closeness (mimicking/anti-mimicking interaction partner (i.e., confederate), the experimenter, one’s romantic partner, best friend, and mother) and two Bayesian ANCOVA models for prosocial behavior (individual donations to charity and willingness to help the mimicking/anti-mimicking interaction partner): Social closeness/Prosocial Behavior (per DV) ~ Mimicry Group + perspective taking + empathic concern + pain tolerance + Mimicry Group * perspective taking * empathic concern * pain tolerance; (all sub-interactions are calculated by JASP; see OSF https://osf.io/f7chd/?view_only=db596702015044cfb9afc1e3f517ec2d annotated JASP file for full model). Bayes factors are transitive, which allowed us to compare the predictive performance of the best model to the model including only being mimicked versus anti-mimicked (Mimicry Group). In the case of the model including only Mimicry Group as best model we compared its predictive performance to the null model.

As listed above, we had hypothesis for each of the five dependent variables of social closeness and the two factors of prosocial behavior. For each model, we present the uncorrected Bayes Factor (BF_10_) and the BF_10_ corrected for multiple comparisons for our hypothesis: Being mimicked leads to an increase in social closeness/prosocial behavior in individuals with high scores on empathy-related traits and increased endorphin release (pain tolerance) as compared to only being mimicked. Correction of the BF_10_ was calculated for five comparisons of dependent variables for social closeness and two comparisons of dependent variables of prosocial behavior. We used the correction following Westfall’s method^72,73^ using a Matlab code (written for statistical tests with one comparison (e.g., Mann–Whitney-U, T-test or post-hoc test after an ANOVA) published on OSF (https://osf.io/twxsk/^74^).

We further report uncorrected additional information on the best model: The BF_M_ indicates the change from prior to posterior odds for each model^75^. For the best models, except null models, we further report additional uncorrected analysis of effects, computing model-averaged results, prior and posterior inclusion probabilities and the inclusion Bayes Factor (BF_incl_) indicating the change of predictive performance of the model if the factor is included to when it is excluded^75^. Prior probability of all models was set to equal. See supplementary material for exploratory Bayesian ANCOVAs including the added between-subject factor *confederate* in the aforementioned models for social closeness, prosocial behaviours and additional rating scales of feelings of sympathy towards, as well as trustworthiness and likeability of the confederate (S3.3) (best models compared to the model including only Mimicry Group).

## Results

### Social closeness

**Figure 1:**
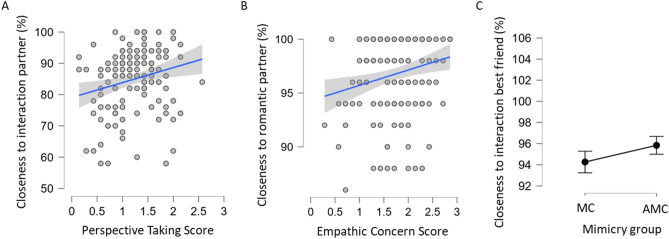
(**A–C)** Experienced social closeness to interaction partner (confederate), best friend and romantic partner in percent (%). Percentage of experienced closeness to (**A**) interaction partner, (**B**) romantic partner in relation to individual EC Score; (**C**) best friend in relation to Mimicry group (mimicry/anti-mimicry condition); Shaded area (**A**,**B**), error bars (**C**): credible interval (95%).

Bayesian ANCOVA was conducted for social closeness for the between-subject factor of being mimicked versus anti-mimicked with the covariates perspective taking, empathic concern, pain tolerance and their interaction: for the *mimicking and anti-mimicking interaction partner* this revealed that the data are 14.493 (BF_10_) times more likely under the best model of perspective taking (BF_M_ = 32.618) than Mimicry Group (BF_10_ = 0.069; BF_M_ = 1.897). Correction for multiple comparison suggests BF_10_ = 6.0032. Prior model probability of the best model with perspective taking of *P*(M) = 0.006 changed to a posterior model probability of the best model of *P* (M|D) = 0.164. This suggests moderate evidence for high individual perspective taking scores to increase social closeness to the (anti) mimicking interaction partner as compared to the model including only Mimicry Group. Model-averaged results indicate that that the data are 4.294 times (BF_incl_) more likely under models including perspective taking than in models without this predictor. The prior inclusion probability for models including PT changed from *P*(incl) = 0.114 to a posterior inclusion probability of *P*(incl|D) = 0.486. For the *experimenter* this revealed that the data are 1.319 (BF_10_) more likely under the best model which is the null model (BF_M_ = 8.223) than the one with only Mimicry Group (BF_10_ = 0.758; BF_M_ = 6.158). Prior model probability of *P*(M) = 0.006 changed to a posterior model probability of *P* (M|D) = 0.0436 for the null model. Correction for multiple comparison suggests BF_10_ = 0.5463. This suggests anecdotal evidence for a null effect of experienced social closeness to the experimenter as compared to a model including only Mimicry Group. For the *romantic partner* the data are 34.483 (BF_10_) times more likely under the best model of empathic concern (BF_M_ = 64.554) than under the model including only Mimicry Group (BF_10_ = 0.029; BF_M_ = 1.349). Correction for multiple comparison suggests BF_10_ = 14.2833. Prior model probability of *P*(M) = 0.006 changed to a posterior model probability *P* (M|D) = 0.280 for the best model. Model-averaged results indicate that the data are about 7.957 times (BF_incl_) more likely under models including empathic concern than in models without this predictor. This suggests strong evidence for high individual empathic concern scores to increase social closeness to the (anti) mimicking interaction partner more than Mimicry Group. For the *best friend* this revealed the data is 2.227 (BF_10_) times more likely under the best model of Mimicry Group (BF_M_ = 19.524) than the null model (BF_10_ = 0.449; BF_M_ = 8.240). Correction for multiple comparison suggests BF_10_ = 0.9225. Prior model probability of the best model including Mimicry Group of *P*(M) = 0.006 to a posterior model probability of *P* (M|D) = 0.105. Model-averaged results indicate that the data are about 2.360 times (BF_incl_) more likely under models including Mimicry group than in models without this predictor. The prior inclusion probability for models including perspective taking changed from *P*(incl) = 0.072 to a posterior inclusion probability of *P*(incl|D) = 0.331. This suggests anecdotal evidence for being anti-mimicked alone to increase social closeness to the best friend more than being anti-mimicked and endorphin release (see Fig. 1C). For *one’s mother* this revealed that the data are 5.319 (BF_10_) times more likely under the best model, which is the null model (BF_M_ = 97.243) than under the model including Mimicry Group (BF_10_ = 0.188; BF_M_ = 12.360). Prior model probability of the null model of *P*(M) = 0.006 changed to a posterior model probability of *P* (M|D) = 0.369. Correction for multiple comparison suggests BF_10_ = 2.2032. This suggests anecdotal evidence for a null effect for the data of experienced social closeness to one’s mother compared to the model with Mimicry Group.

### Prosocial behaviour

**Figure 2:**
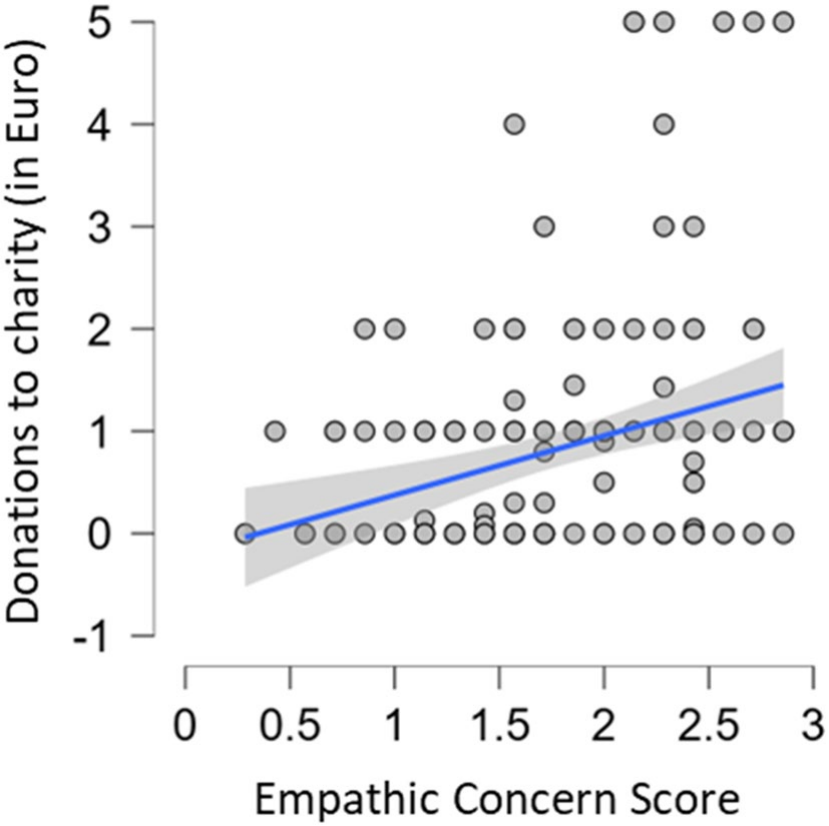
Amount of individual donations to charity (in Euro) in relation to empathic concern scores. Shaded area: credible interval (95%).

**Figure 3:**
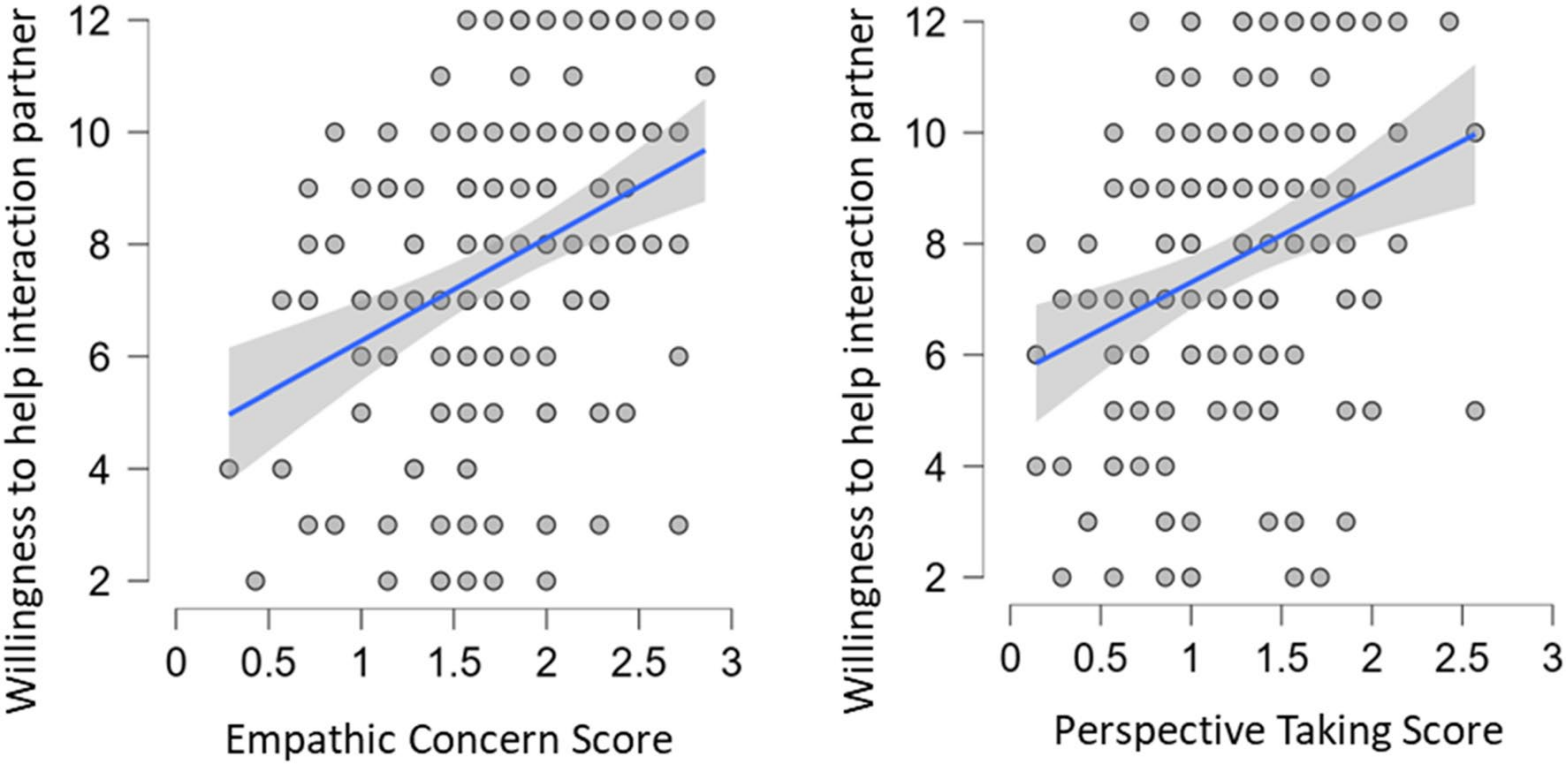
Willingness to help (anti-)mimicking interaction partner (confederate) in relation to perspective taking (left) and empathic concern scores (right). Shaded area: credible interval (95%).

The Bayesian ANCOVA by Mimicry group with perspective taking, empathic concern, pain tolerance and the defined interactions revealed: For *individual donations* (Fig. 2) that the data are 500 (BF_10_) times more likely under the best model including empathic concern (BF_M_ = 69.711) than the model including Mimicry Group (BF_10_ = 0.002; BF_M_ = 0.113). Prior model probability of *P*(M) = 0.006 changed to a posterior model probability of the model with empathic concern of *P* (M|D) = 0.296. Correction for multiple comparison suggests BF_10_ = 290. This suggests extreme evidence of high individual perspective taking scores to increase individual donations compared to the model including only Mimicry Group (see Fig. 2). Model-averaged results indicate that that the data are about 69.561 times (BF_incl_) more likely under models including empathic concern than in models without this predictor. The prior inclusion probability for models including empathic concern changed from *P*(incl) = 0.771 to a posterior inclusion probability of *P*(incl|D) = 0.986. For the *willingness to help the participant* (Fig. 3) this revealed that the data are 105 030 (BF_10_) times more likely under the best model including the two main effects of empathic concern and perspective taking (BF_M_ = 67.522) than Mimicry Group (BF_10_ = 9.521*10^–6^; BF_M_ = 4.570*10^–4^). Prior model probability of *P*(M) = 0.006 changed to a posterior model probability of the best model with empathic concern and perspective taking of *P*(M|D) = 0.289. Correction for multiple comparison suggests BF_10_ = 61 690. Model-averaged results indicate that that the data are about 243.502 times (BF_incl_) more likely under models including empathic concern than in models without this predictor. The prior inclusion probability for models including empathic concern changed from *P*(incl) = 0.114 to a posterior inclusion probability of *P*(incl|D) = 0.644. The data are 4.952 times more likely under models including perspective taking than without it. The prior inclusion probability for perspective taking changed from *P*(incl) = 0.114 to a posterior inclusion probability of *P*(incl|D) = 0.566. This suggests extreme evidence that high individual empathic concern and perspective taking scores increase individual donations compared to the model including only Mimicry Group (see Fig. 3).

## Discussion

We investigated the influence of being mimicked versus anti-mimicked on social closeness and prosocial behaviour in relation to empathy-related traits, a proxy for endorphin uptake and their interactions as compared to being mimicked or anti-mimicked alone (model including only Mimicry Group). Results suggest moderate evidence that high individual scores on perspective taking increase social closeness to the (anti-) mimicking interaction partner (Fig. 1A). They suggest strong evidence that high individual scores on empathic concern increase social closeness to the romantic partner (Fig. 1B) as compared to the model including only Mimicry Group (Fig. 1C). Results further suggest anecdotal evidence that being anti-mimicked increased social closeness to the best friend as compared to the null model. No effects on experienced social closeness for the experimenter and one’s mother were found (null effect).

Results showed extreme evidence that empathy-related traits increase prosocial behaviour as compared to the model including only Mimicry Group. Results suggest extreme evidence that high individual empathic concern scores increase donations to a charity (Fig. 2) and that high scores on both individual empathic concern and perspective taking increase the willingness to help the (mimicking/anti-mimicking) interaction partner (Fig. 3). These results suggest empathy-related traits as the core determinants for experienced social closeness (anti-mimicking/mimicking interaction partner, romantic partner) and prosocial behaviour (donations and willingness to help).

### Empathy-related traits: social closeness

In the present study, empathy-related traits are the primary determinant for experienced social closeness to the anti-mimicking/mimicking interaction partner and to one’s romantic partner. This suggests that high individual scores of perspective taking may increase social closeness to the new interaction partner. Indeed, perspective taking has been proposed as a vital strategy for the establishment of social bonds^6^, facilitating smooth and rewarding relationships^76^. Heightened perspective taking may enable self-other overlap of mental representations between interaction partners, facilitating social bonding. This self-other overlap may be driven by seeing more of the self in the other, and an inclusion of the self in the other^6^.

Closeness to one’s romantic partner seems to increase with high individual scores of empathic concern. Empathic concern^8,36^ is an inherently other-oriented emotional response^8^, concerned with improving the other’s state^9^, as mentioned above. The present results suggest that this focus on the well-being of another is the driving force behind the subjective closeness felt towards one’s romantic partner. Previous reports have suggested that adults in romantic relationships show less mimicry to an attractive confederate^77^, moderated by their reported love for their romantic partner^78^. The present study suggests that the display of temporally contingent behaviour by a new interaction partner did not influence how close participants felt to their romantic partner. This was different for experienced closeness to the best friend. There was anecdotal evidence that closeness to the best friend increased when being anti-mimicked (Fig. 1C). Potentially being anti-mimicked reduced subjective closeness to the best friend, as if being reminded of the special close relationship quality with the best friend in comparison with a contingent, but topographically unaligned new acquaintance. Whereas being mimicked with both temporally contingent and topographically isomorph postures could allow establishment of a new bond with the new interaction partner (i.e., confederate), thus reducing subjective closeness in comparison to the best friend.

Both the perceived closeness to one’s mother and the experimenter were not influenced by either Mimicry Group, empathy-related traits, or endorphin release. Family relations may be more stable to external social influences than self-chosen ones^79^. Closeness to the experimenter was not modulated by any of the variables of interest. This may provide support against the generalizability of mimicry behaviour to new acquaintances if the interactional behaviour was not contingent (whether topographically iso- or heteromorph). It also speaks against generalizability of high empathy-related traits automatically inducing increased closeness to unknown others with whom one has not interacted with temporal contingency, nor aligned in postures (as the experimenter). Yet, as shown in the supplementary material, the model including confederate is better than the null model in explaining the data of the model on social closeness to the experimenter (anecdotal evidence; see S3.3, Figure S2).

### Empathy-related traits: prosocial behaviour

Results suggest extreme evidence that individual empathy-related traits increase prosocial behaviour as compared to the model including only Mimicry Group. High individual scores on empathic concern increase donations to charity (“Doctors Without Borders”) and, together with individual perspective taking scores, increase willingness to help the interaction partner. As mentioned above, empathy may have primarily evolved to support prosocial behaviour^11^. Several accounts have implicated empathic concern in costly prosocial behaviour, both in a highly distressing social interaction paradigm, including decisions between financial self-gain and the physical well-being of the interaction partner^80^ and when taking over another’s pain^81^. This seems in line with experiments in a virtual environment: participants who risked their (virtual) life to save somebody else in danger also exhibited increased empathic concern^82^.

Perspective taking is also, along with empathic concern, involved in driving prosocial behaviour regarding the new interaction partner (i.e., the confederate; Fig. 3). Dissociable brain networks have predicted donations when participants reported empathic feelings (i.e., affective empathy) for a charity’s goal beforehand, as compared to taking the perspective (i.e., cognitive perspective taking) of the charity’s cause^83^, thus highlighting different mechanisms behind each trait in relation to prosocial behaviour. A differentiation of neural signatures on prosocial behaviour based on perspective taking or empathic concern was also reported in a recent meta-analysis^84^. Also the neural and behavioural efficiency of prosocial learning is predicted by empathy-related traits^85^.

### Temporal contingency in mimicry behaviour

Our results suggest, that, apart from weak evidence for social closeness to the best friend, social closeness to the new interaction partner (i.e., confederate) and the romantic partner, as well as prosocial behaviour, are driven by empathy-related traits after an interaction of contingent postural alignment (even if topographically heteromorph) in both the anti-mimicry and mimicry condition. Temporal contingency in interactional behaviour could be sufficient in creating an overlap of self- and other generated movements in the establishment of a new relationship. A temporally contingent, thus reliably reciprocal, link between the perception and execution of interaction behaviour may create an interactive sensory overlap of movement, tuning the brain into an optimal state of temporal prediction of the other’s motor behaviour (i.e., predictive coding)^86^. This optimized prediction may allow facilitation of behavioural coordination^86,87^, leading to a feeling of self-other overlap^88^. Regarding the present results, an interaction with a stranger, who aligns behaviour and postures in a temporally contingent manner (i.e., both the mimicking and anti-mimicking participant), may lead to an optimal prediction state if the participant is scoring high on empathy-related traits. As such, empathy-related traits may influence the sensitivity of perception to being mimicked in a temporally contingent, even if not topographically isomorph, manner, but this awaits further investigation.

### Endorphin release

We did not find any effects of endorphins on social closeness in relation to being mimicked or anti-mimicked and empathy-related traits. Neither was endorphin release influenced in general by whether participants were mimicked versus anti-mimicked (see supplementary material). This is in contrast to findings on synchronous behaviour. Synchronous movement, such as dancing^15^, joint laughter^13,51^ or singing^55^ may create more exertion and evoke a stronger reaction of the endorphin system^58^, resulting in increased feelings of cohesion than a one-shot sitting encounter of postural alignment. Genetic variation in the mu-opioid receptor gene (Opioid receptor Mu 1, OPRM1) may be mainly responsible for social disposition. It is associated with dyadic relationship quality, but also community integration^12^. Other neuropeptides or neurohormones, not investigated in this study, could also be involved in modulating the positive effects of being mimicked^12^. While beta endorphins may play a role in the maintenance of stable long-term relationships, serotonin, oxytocin and dopamine may be associated with their onset^44^. Yet, while the mu-opioid receptor (MOR) is associated with secure attachment, the same study did not find this association for serotonin^89^. In a genetic analysis, serotonin has only been found associated with the wider network, while oxytocin seems associated with dyadic relationships^12^. It may be involved in various forms of social alignment (rev.^90^) and may increase emotional facial mimicry^91^ (but see^92^). The release of dopamine during reward may likewise be involved in modulating the positive effects of synchrony^90^. Whether or which other neuropeptides or neurohormones are involved in creating beneficial effects of being mimicked, or at which stage, remains a topic of investigation. Although we did not find evidence that being mimicked influences endorphin release, this does not preclude its involvement in repeated mimicry interaction or the maintenance of stable relationships in interaction with empathy-related traits.

## Limitations

For our study we used a well-powered sample to detect potential (null-) effects (see supplementary material). Nonetheless, some limitations remain.

Our choice of the anti-mimicry condition as control condition was based on previous findings^17^. We aimed to keep movement constant across conditions, to extend seminal studies (e.g.,^1,3^) presenting a non-moving confederate as control condition. An inactive, passive control condition may not be the most ideally suited, given the different level of motor activity during interaction, which may itself negatively influence social outcomes. While participants’ posture changes are constantly matched with reciprocal movement when being mimicked, there would be no movement at all in response to posture changes in an inactive, passive control condition. A non-engaging, passive interaction partner may then themselves even elicit negative reactions from the participant, thus rendering being mimicked particularly favorable. Yet, anti-mimicry could have itself provided a type of mimicry induction. Temporally contingent motor alignment may be sufficient to establish positive social effects. A different control condition of random movement by the confederate, both in time and moving body parts, may have been preferable. However, we aimed to ensure that movement frequency was constant across conditions. A replication study including a completely random condition could shed light on the effects of temporally contingent interpersonal behaviour (i.e., anti-mimicry).

Due to technical problems, the video recordings of most participants could not be used for video analysis of the (anti-) mimicking behaviour of the confederate, to identify e.g., potential smiles or eye-contact. While this is major limitation, we provide additional analysis, adding the between subject factor of “confederate” in the analysis in the supplementary material (S3.2; Bayesian ANCOVA including between-subject factors Mimicry Group and confederate, covariates empathy-related traits and pain tolerance). We also performed the same additional Bayesian ANCOVA on the feeling of sympathy for the confederate, their likeability and how trustworthy they appeared. Adding the factor “confederate” does not change the results of models presented in the main text with the best predictive performance on social closeness and prosocial behaviour being driven by empathy-related traits (models compared to model including only Mimicry Group; see supplementary material S3.2, and annotated JASP file on OSF https://osf.io/f7chd/). However, there was weak evidence that social closeness to the experimenter increased when interacting with confederate 2 as compared to confederate 1 (see S3.2, Fig. S2). Participants felt more sympathy towards confederate 1 than confederate 2 when they scored high on perspective taking (see S3.2, Fig. 3). Additional results (see S3.2) show that ratings of trustworthiness of both confederates increased with high scores on perspective taking; no effect was found for likeability of the confederates (see S3.2). The instruction to confederates to show natural, polite, behaviour refraining from smiles, eye contact, laughter, or touch is in line with seminal studies (e.g.,^1^). The goal of this instruction was to test solely the effects of being mimicked/anti-mimicked without other confounding social cues that may signal affiliation and vary between confederates and participants.

We used a between-subject, instead of a within-subject design, to avoid cross-over effects between two sessions of the same experiment. In a within-subject design, being mimicked versus anti-mimicked and the computerized tasks would have already been familiar, altering results.

Our rating scale question of “willingness to help the other participant”, who was in reality a confederate, was unspecific to the kind of helping behaviour that would be offered. A more nuanced rating scale targeting different helping scenarios would have given more specific information.

Endorphin release was measured indirectly via pain tolerance in a wall-sit ski exercise. While comfortable at first, this task quickly becomes excruciatingly painful, and few people can maintain it for more than about 90 secs. While differences in participants’ endurance level may influence the baseline measure of holding the position, we were only interested in the change measure of pain tolerance before and after the mimicry/anti-mimicry condition. The wall-sit ski exercise is less susceptible to subjective bias than other indirect measures of endorphin release, as e.g., the “pressure cuff test” in which pain tolerance is measured using inflation of a blood pressure cuff^66^. Alternative assays involve either a psychopharmacological intervention using an endorphin blocker or the use of PET scans. However, the mu-opioid system has been reliably demonstrated in pain modulation^42,93,94^, and pain tolerance measures have been shown to trigger the endorphin system^57,58^. Clearly, other neurotransmitters, such as dopamine, serotonin, oxytocin or vasopressin, may also be involved in regulating social behaviour (e.g.,^12^), some of them also have analgesic effects (e.g.,^47,48^), and may work in concert with the opioid system^40^. Examination of the involvement of these other neurotransmitters would pay dividends.

Regardless of these limitations, we present a well-powered assessment of the beneficial social effects of empathy-related traits on social closeness and prosocial behaviour. Our results suggest that empathy-related traits are the main driver of positive social effects as long as interaction is reliably reciprocal (temporally contingent).

## Applications

This study has a number of applications. Interpersonal motor alignment could be imagined on combination of a temporal and topographic dimensions from complete (100%) temporal and topographic overlap (i.e., synchronous behaviour) to no interactive motor alignment on either dimension (0% on both). Likewise, these dimensions may be self-sufficient and temporal rhythmic overlap without any (visible) topographic alignment may readily elicit feelings of social closeness. Synchronous singing for example exhibits fast social bonding effects^55^. This represents a multimodal synchronous behaviour engaging temporal, rhythmic information, and synchronization of sounds, respiration and heart rate, without strong visible synchronization of limb movement or postural changes. Future research could dissect these dimensions more carefully to determine at which point interactive motor alignment yields the most beneficial versus no more social effects.

The present study highlights the influence of empathy-related traits on social benefits. Targeted use of empathy training may aid various therapeutic interventions, especially in a world that has gone online during the recent pandemic, to foster deeper social connection. During the pandemic, children, adolescents and senior citizens have been particularly adversely affected by social isolation^95^. Interactive motor alignment is under-investigated in adolescents^21^, who rely heavily on peer exchange. Likewise, older adults suffer from isolation with risk for depression and anxiety^96^. Empathy training paired with contingent interaction, maybe presented in the form of online video games for adolescents, may strengthen social resilience.

## Conclusion

Our results extend previous findings in an important respect: the results challenge the prominent findings of increased social closeness and prosocial behaviour after being mimicked. They suggest that as long as interaction is temporally contingent, high individual empathy-related traits increase social closeness to the new anti-mimicking/mimicking interaction partner and the romantic partner, but especially prosocial behaviour. Our results encourage re-examination of the role of topographically isomorph mimicry in fostering social closeness and prosocial behaviour. This may have important implications for interventions targeting social resilience programs to increase well-being, potentially even in a virtual environment.

## Supplementary Information

Supplementary Information.

## Data Availability

The datasets generated and/or analysed during the current study are available in the Open Science Framework (OSF) repository https://osf.io/f7chd/.
