# Supplementary material for: Only empathy-related traits, not being mimicked or endorphin release, influence social closeness and prosocial behavior
Source: Sci Rep. 2023 Mar 11;13:4072. doi: 10.1038/s41598-023-30946-9 (PMC10008555; doi:10.1038/s41598-023-30946-9)
Supplement: Supplementary file 1 — Supplementary Information. [file 41598_2023_30946_MOESM1_ESM.docx]

**Supplementary Material**

**Only empathy-related traits, not being mimicked or endorphin release, influence social closeness and prosocial behavior**

1. Pilot study: The effects of being mimicked on endorphin release

The pilot study included thirty-nine female participants, of which twenty were in the Mimicry and nineteen in the Anti-Mimicry condition (Mean age: M = 24.59 years, SD = 4.36). We used changes in pain threshold in a wall sit ski exercise ^1–3^ as proxy for changes in endorphin release. The wall-sit ski exercise was carried out before and after participants were being mimicked or anti-mimicked by an ostensible other participant, who, in reality, was a female confederate (see main text for details on wall-sit ski exercise and Mimicry/Anti-Mimicry Induction)

We hypothesized that being mimicked would increase endorphin release, measured with a proxy of pain threshold in the wall-sit ski exercise as compared to being anti-mimicked.

For this we conducted an independent t-test comparing the change in pain threshold between Mimicry groups. Also, we conducted Bayesian independent t-test investigating this directed hypothesis.

- 1. ***Results***
     1. ***ΔEndorphin release***
        1. *Frequentist analysis*

An independent t-test comparing the change of T2 minus T1 by mimicry group reveals with a directed hypothesis of The change of T2-T1 being greater for Mimicry than Anti-Mimicry revealed significant difference by trend between Mimicry groups (t(37)= 1.611, p = .058, Cohen’s d = .516).

- - - 1. *Bayesian independent t-test*

The Bayesian independent samples t-test revealed BF+0 = 1.578, with a BF0+ = 0.634, (95percent CI: = (0.040, 1.038), thus providing anectodal evidence for the directed H1 over the H0 (see supplementary material for Bayes factor robustness check).

Frequentist analysis showed a significant result by trend with an increase in pain threshold in the Mimicry condition. Bayesian analysis suggested anectodal evidence for the H1 that being mimicked increases pain threshold compared to the Anti-Mimicry condition.

- 1. ***Sample size calculations***

Sample size for the main study was calculated using the data from the change in endorphin release before (T1; in milliseconds) and after (T2) being mimicked or anti-mimicked using G*Power (http://www.gpower.hhu.de/;^4,5^).

The change in endorphin release was calculated as the difference between T2 minus T1. Results showed a change in endorphin release for the Mimicry condition with a mean of M=-416 ms (SD = 28532.10) and for the Anti-Mimicry condition with a mean of M = -19137.37 ms (SD = 42953.45). This revealed an effect size of Cohen’s d of 0.513) for an independent t-test comparing the change in endorphin release between Mimicry group. Expected power (1-β) was set to .95 in G*Power. G*Power calculated a required sample size of 83 participants per Mimicry group (in total 166) (see supplementary material section 1.1 for G*Power protocol). The sample size was then set to 180 in total (90 participants in a between-group experiment) to account for potential data loss.

1. **Images of the International Affective Picture System (IAPS) used for the Mimicry/Anti-Mimicry induction (see also supplementary material in** ^6^**)**

| **Description** | **IAPS** | **valmn** | **valsd** | **aromn** | **arosd** | **dom1**  **mn** | **dom1**  **sd** | **dom2**  **mn** | **dom2**  **sd** | **set** |
| --- | --- | --- | --- | --- | --- | --- | --- | --- | --- | --- |
| NeuMan | 2102 | 5.16 | 0.96 | 3.03 | 1.87 |  |  | 5.80 | 1.77 | 16 |
| NeuWoman | 2104 | 4.42 | 1.09 | 3.11 | 1.84 |  |  | 5.45 | 1.84 | 15 |
| Farmer | 2191 | 5.30 | 1.62 | 2.23 | 2.14 |  |  | 5.80 | 2.00 | 14 |
| Butcher | 2235 | 5.64 | 1.27 | 3.36 | 1.92 |  |  | 5.83 | 1.91 | 13 |
| Girl | 2320 | 6.17 | 1.51 | 2.90 | 1.89 | 6.66 | 1.81 |  |  | 10 |
| Secretary | 2383 | 4.72 | 1.36 | 3.41 | 1.83 | 5.75 | 1.89 |  |  | 12 |
| Factoryworker | 2393 | 4.87 | 1.06 | 2.93 | 1.88 |  |  | 5.83 | 2.06 | 13 |
| Musician | 2487 | 5.20 | 1.80 | 4.05 | 1.92 | 5.81 | 2.09 |  |  | 12 |
| Harvest | 2515 | 6.09 | 1.54 | 3.80 | 2.12 | 6.52 | 1.73 |  |  | 11 |
| Propeller | 2575 | 5.46 | 1.15 | 4.16 | 2.10 | 6.11 | 1.79 |  |  | 11 |
| City | 2594 | 6.05 | 1.31 | 3.84 | 1.98 |  |  | 5.78 | 1.85 | 15 |
| Shopping | 27451 | 5.31 | 1.08 | 3.26 | 1.96 |  |  | 6.29 | 1.96 | 14 |
| Tourist | 2850 | 5.22 | 1.39 | 3.00 | 1.94 | 5.87 | 1.97 |  |  | 10 |
| Flowers | 5731 | 5.39 | 1.58 | 2.74 | 1.95 | 6.13 | 1.77 |  |  | 8 |
| Office | 7550 | 5.27 | 1.40 | 3.95 | 1.91 | 5.22 | 2.26 |  |  | 3 |
| Golfer | 8311 | 5.88 | 1.67 | 3.57 | 2.35 | 5.80 | 1.74 |  |  | 6 |
| Bicyclist | 5875 | 6.03 | 1.43 | 3.29 | 2.12 | 6.19 | 1.91 |  |  | 12 |
| Men | 2593 | 5.80 | 1.34 | 3.42 | 1.84 |  |  | 5.91 | 1.69 | 16 |
| Picnic | 2560 | 6.34 | 1.53 | 3.49 | 2.07 | 6.06 | 1.85 |  |  | 10 |
| Market | 2597 | 5.61 | 1.26 | 4.09 | 2.10 |  |  | 5.18 | 1.83 | 16 |

**Table S1: Images of the International Affective Picture System (IAPS) used for the Mimicry/Anti-Mimicry induction.** Table contains names (Description), corresponding number (IAPS), mean of valence ratings (valmn) with standard deviation (valsd), mean of arousal ratings (aromn) with standard deviation (arosd), mean ratings of dominance 1 (dom1mn) with standard deviation (dom1sd) and mean ratings of dominance 2 (dom2mn) with standard deviation (dom2sd) and the number of picture set (set) of used IAPS images for the (Anti-) Mimicry induction; (Assessment of values with different version of Self-Assessment Manikin ^7^).

1. **Additional results**

***Change in pain tolerance.*** Time in the wall-sit ski exercise was measured in seconds before (T1) and after (T2) participants were being mimicked or anti-mimicked. We conducted Bayesian analysis of covariance (ANCOVA) for T2 by Mimicry group controlling for T1 with perspective taking (PT), empathic concern (EC) and their interactions as covariates. ***Additional rating scales:*** Additional rating scales on sympathy feelings towards the interaction partner (i.e., confederate) and her likeability were conducted. See Rauchbauer et al. (2020)^6^ for rating on social closeness to interaction partner. We conducted Bayesian analysis of covariance (ANCOVA) for each scale by Mimicry group controlling for perspective taking (PT), empathic concern (EC), the ΔT2-T1 and their interactions as covariates.

- 1. ***Effects of Mimicry/Anti-Mimicry and trait empathy covariates on pain tolerance (increase in endorphin release)***

Bayesian ANCOVA on change in pain tolerance (timepoint 2, T2) by Mimicry group with the baseline time (timepoint, T1), perspective taking, empathic concern as covariate and their interactions revealed that the data are 4.003e+31 times more likely under the best model with T1 than the null model. Prior model probability of the model with T1 of P(M) = 0.006 changed to a posterior model probability of P (M|D) = .692 (see Figure 1A). This suggests extreme evidence for the model with T1. Furthermore, the data is 6.194 times more likely under T1 than the next best model including T1 and Mimicry group. The model T1 and Mimicry group is still 2.602/4.003e+31 times more likely than the null model (see Figure 1B).

Model-averaged results indicate that the data are 2.696e+31 times (BF(incl)) more likely under model including T1 than in models without this predictor. The prior inclusion probability for models including T1 changed from P(incl) =0.114 to a posterior inclusion probability of P(incl|D)= .987. The data are only about 0.386 times (BF(incl)) more likely under models including Mimicry group than in models without this predictor. The prior inclusion probability for models including Mimicry group changed from P(incl) =0.114 to a posterior inclusion probability of P(incl|D)=0.276.


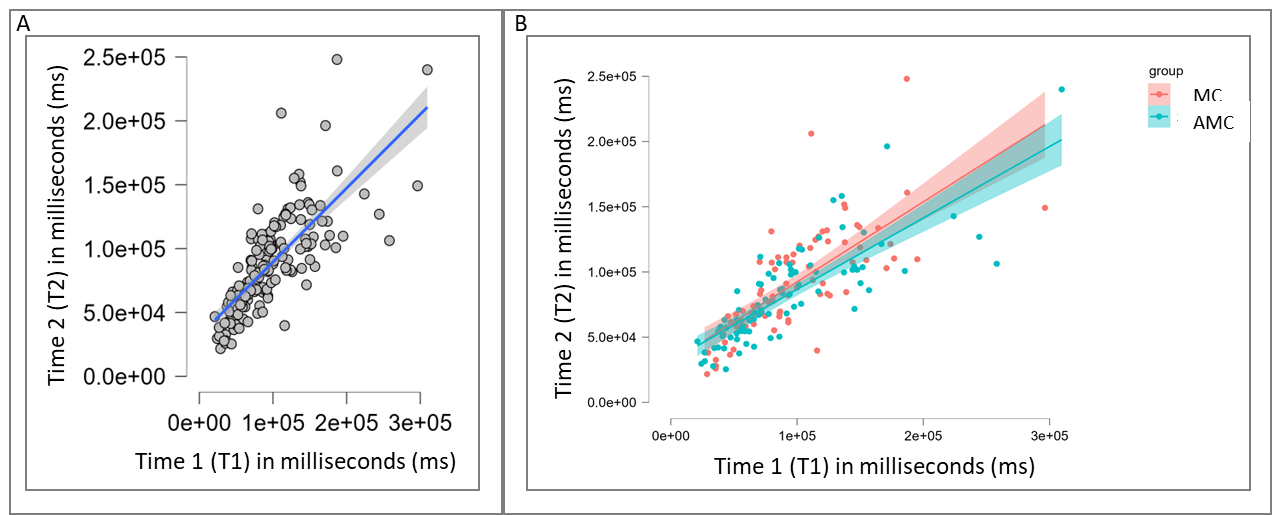


**Figure S1: Change in Time 2 (T2) by baseline time measurement (Time 1, (T1)) in milliseconds (ms) (A), and by group (B).** MC stands for Mimicry Condition, AMC for Anti-Mimicry Condition. Shaded area: credible interval (95%).

**Discussion.** Results showed that the data was best predicted by the model including baseline pain tolerance alone. As seen in Figure 1B, the second-best model including T1 and Mimicry Group may suggest a direction of pain tolerance increasing in the Mimicry condition, yet the mode including T1 was found as the best predictor of the data, while in the second-best model the confidence intervals overlap.

Our findings, contrary to the findings of synchronous interaction promoting endorphin release, could be due to a difference in baseline pain tolerance measures due to a variation in Mu opioid receptor (MOR) availability, frequency of doing sports, or personality factors. Synchronous movement, such as dancing ^8^, joint laughter ^1,9^ or singing ^10^ may create more exertion and evoke a stronger reaction of the endorphin system ^11^, resulting in increased feelings of cohesion than a one-shot sitting encounter of postural alignment. Using a control condition with not temporal contingent motor alignment may give a clearer picture on whether also being mimicked may elicit endorphin release, as suggested by the second-best model.

- 1. ***Data analysis and results social closeness, prosocial behavior, and additional ratings of confederate (likeability, sympathy, trustworthiness) including confederate as additional between-subject factor***

**3.2.1 Data analysis.** Bayesian analyses were conducted using JASP (https://jasp-stats.org/; van Doorn et al., 2019). As in the main text, we investigated the social closeness (in percent) and prosocial behavior for each dependent variables separately in a Bayesian ANCOVA with the covariates of perspective taking, empathic concern, pain tolerance (proxy for endorphin release) and confederate in separate models to evaluate each of their predictive performance: Social closeness/Prosocial Behavior/Confederate Ratings (per dependent variable) ~ Mimicry Group + perspective taking + empathic concern + pain tolerance + confederate; all sub-interactions are calculated (see OSF <https://osf.io/f7chd/?view_only=db596702015044cfb9afc1e3f517ec2d> annotated JASP file for full model; We did not model interactions, as this overloaded the model). This yielded five separate Bayesian ANCOVAs for social closeness, two for prosocial behavior, three for confederate ratings that we compare, as in the main text, to the model including only Mimicry Group. We did not expect an effect of the between-subject factor of confederate on the predictive power of the models. For each, we present the uncorrected Bayes Factor (BF_10_), and corrected for multiple comparisons ((social closeness: 5 comparisons, prosocial behavior: 2 comparisons, ratings: 3 comparisons), which was corrected with Westfall’s method^13,14^ using a Matlab code published on (<https://osf.io/twxsk/>)^[[1]](#footnote-1)^ ^15^; see also main text). We also report the BF_M_ indicating the change from prior to posterior odds for each model ^16^. Prior probability of all models was set to equal.

**3.2.2 Results**

**Social closeness.**  Bayesian ANCOVA was conducted for social closeness by Mimicry group (Mimicry/ Anti-Mimicry condition) with perspective taking, empathic concern, pain tolerance and confederate: for the ***mimicking and anti-mimicking interaction partner*** this revealed that the data are 7.086 (BF_10_) times more likely under the best model of perspective taking (BF_M_ = 8.940) than Mimicry Group (BF_10_ = 0.274; BFM = 2.104). Correction for multiple comparison suggests BF_10_ = 2.935. Prior model probability of the best model with perspective taking of P(M) = 0.025 changed to a posterior model probability of the best model of P (M|D) = .187. This suggests moderate evidence that high individual empathic concern scores increase social closeness to the anti-mimicking/mimicking interaction partner more than Mimicry Group alone. For the ***experimenter*** this revealed that the data are 1.497 (BF10) more likely under the best model with confederate (BF_M_ = 3.826) than the model including only Mimicry Group (BF_10_ = 0.668, BF_M_ = 2.465). Prior model probability of P(M) = 0.025 changed to a posterior model probability of P (M|D) = .089 for the best model including confederate. Correction for multiple comparison suggests BF_10_ = 0.620. This suggests anectodal evidence for a null effect. For the ***romantic partner*** the data are 21.277 (BF_10_) times more likely under the best model of empathic concern (BFM = 21.795) than under the model including only Mimicry Group (BF_10_ = 0.047; BFM = 0.667). Correction for multiple comparison suggests BF_10_ = 8.813. Prior model probability of P(M) = 0.025 changed to a posterior model probability P (M|D) = 0.359 for the best model. This suggests moderate evidence that high individual empathic concern scores increase social closeness to the romantic partner more than Mimicry Group alone. For the ***best friend*** this revealed the data is 1.799 (BF_10_) times more likely under the best model of Mimicry Group (BFM = 13.169) than the null model (BF_10_ = 0.556; BF_M_ = 6.371). Correction for multiple comparison suggests BF_10_ = 0.745. Prior model probability of the best model including Mimicry Group of P(M) = 0.025 to a posterior model probability of P (M|D) = 0.252. This suggests anectodal evidence that being anti-mimicked increases social closeness to the best friend (see Figure 1C). For ***one’s*** ***mother*** this revealed that the data are 5.376 (BF_10_) times more likely under the best model, which is the null model (BF_M_ = 18.768) than under the model including Mimicry Group (BF_10_ = 0.186; BF_M_= 2.510). Prior model probability of the null model of P(M) = 0.025 changed to a posterior model probability of P (M|D) = 0.325. Correction for multiple comparison suggests BF_10_ = 2.227. This suggests anectodal evidence for a null effect.


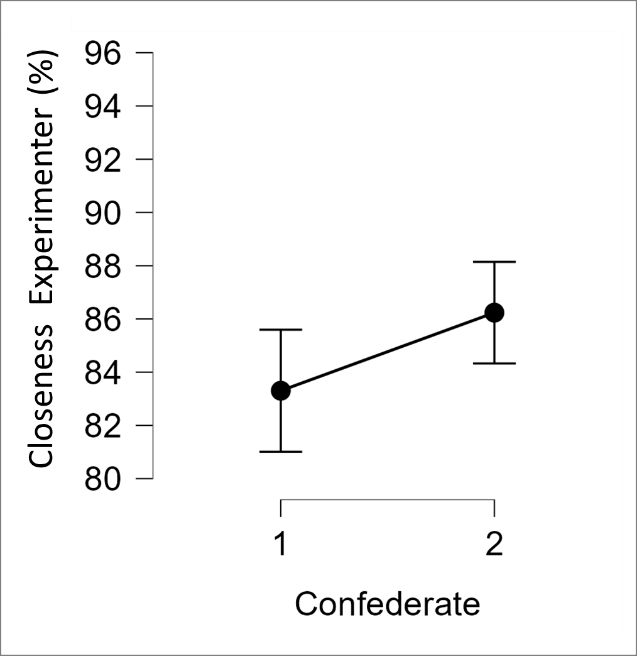


**Figure S2: Experienced social closeness to experimenter (%).** Percentage of experienced closeness to experimenter in interaction with confederate 1 or 2; error bars (C): credible interval (95%).

**Prosocial behavior*.*** The Bayesian ANCOVA by Mimicry group with perspective taking, empathic concern, pain tolerance and the defined interactions revealed: For ***individual donations*** (Fig. 2) that the data are 142.857 (BF_10_) times more likely under the best model including empathic concern (BF_M_ = 17.905) than the model including Mimicry Group (BF_10_ =0.007; BF_M_= 0.080). Prior model probability of P(M) = 0.025 changed to a posterior model probability of the model with empathic concern of P (M|D) = 0.315. Correction for multiple comparison suggests BF_10_ = 83.914. This suggests extreme evidence that high individual perspective taking scores increase individual donations compared to the model including only Mimicry Group (see Figure 2). For the ***willingness to help the participant*** (Fig. 3) the data are 7704.2 (BF_10_) times more likely under the best model including empathic concern (BF_M_ = 20.501) than the model including Mimicry Group (BF_10_ = 1.298*10^-4^; BF_M_= 0.002). Correction for multiple comparison suggests BF_10_ = 4525.5. Prior model probability of P(M) = 0.025 changed to a posterior model probability of the model with empathic concern of P (M|D) = 4.471*10^-5^. This suggests extreme evidence that high individual empathic concern and perspective taking scores increase the willingness to help the anti-mimicking/mimicking interaction partner compared to the model including only Mimicry Group (see Figure 3).

**Rating scale likeability.** Bayesian ANCOVA on the rating scale for likeability of the interaction partner by Mimicry group (Mimicry/ Anti-Mimicry condition) with perspective taking, empathic concern, pain tolerance and confederate. This revealed that the data are 5.236 (BF_10_) times more likely under the best model which is the null model (BF_M_ = 9.707) than Mimicry Group (BF_10_ = 0.191; BF_M_ = 1.541). Correction for multiple comparison suggests BF_10_ = 3.076. Prior model probability of the model with T1 of P(M) = 0.025 changed to a posterior model probability of P (M|D) = .199; This suggests strong evidence that high scores on Perspective Taking increase likeability of the confederate as compared to only Mimicry Group.

**Rating scale sympathy.** Bayesian ANCOVA on the rating scale for sympathy feelings for the interaction partner by Mimicry group (Mimicry/ Anti-Mimicry condition) with perspective taking, empathic concern, pain tolerance and confederate. This revealed that the data are 9.901 (BF_10_) times more likely under the best model of confederate and perspective taking (BF_M_ = 8.725) than Mimicry Group (BF_10_ = 0.101; BF_M_ = 0.018). Correction for multiple comparison suggests BF_10_ = 5.816. Prior model probability of the model with T1 of P(M) = 0.025 changed to a posterior model probability of P (M|D) = .183. This suggests strong evidence that high scores on Perspective Taking increase likeability of the confederate as compared to only Mimicry Group.

**Rating scale trustworthiness.** Bayesian ANCOVA on the rating scale for how trustworthy the interaction partner appears by Mimicry group (Mimicry/ Anti-Mimicry condition) with perspective taking, empathic concern, pain tolerance and confederate. This revealed that the data are 6.410 (BF_10_) times more likely under the best model of perspective taking (BF_M_ = 7.259) than Mimicry Group (BF_10_ = 0.156; BF_M_ = 981). Correction for multiple comparison suggests BF_10_ = 3.760. Prior model probability of the model with T1 of P(M) = 0.025 changed to a posterior model probability of P (M|D) = .157. This suggests strong evidence that high scores on Perspective Taking increase likeability of the confederate as compared to only Mimicry Group. This suggests strong evidence that high scores on perspective taking, and empathic concern increase likeability of the confederate as compared to only Mimicry Group.

***
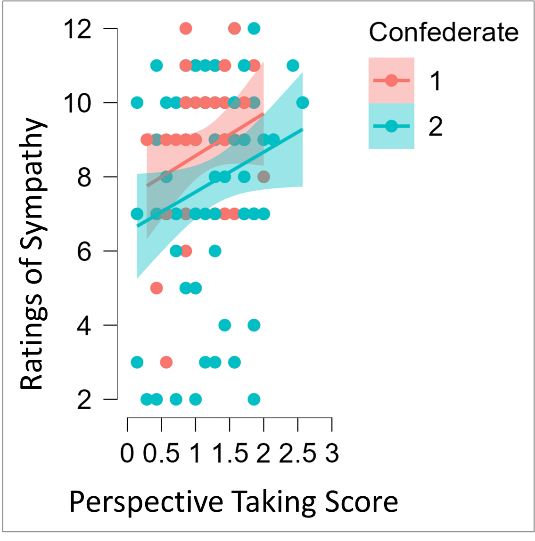
***

**Figure S3: Additional ratings of feelings of sympathy towards confederates.** Additional ratings of feelings of sympathy towards confederate 1 and 2 in relation to individual perspective taking scores; Shaded area: credible interval (95%).

**References**

1. Dunbar, R. I. M. *et al.* Social laughter is correlated with an elevated pain threshold. *Proc. R. Soc. London B Biol. Sci.* rspb20111373 (2011).

2. Harrison, F., Sciberras, J. & James, R. Strength of social tie predicts cooperative investment in a human social network. *PLoS One* **6**, e18338 (2011).

3. Madsen, E. A. *et al.* Kinship and altruism: A cross‐cultural experimental study. *Br. J. Psychol.* **98**, 339–359 (2007).

4. Faul, F., Erdfelder, E., Lang, A.-G. & Buchner, A. G* Power 3: A flexible statistical power analysis program for the social, behavioral, and biomedical sciences. *Behav. Res. Methods* **39**, 175–191 (2007).

5. Faul, F., Erdfelder, E., Buchner, A. & Lang, A.-G. Statistical power analyses using G* Power 3.1: Tests for correlation and regression analyses. *Behav. Res. Methods* **41**, 1149–1160 (2009).

6. Rauchbauer, B., Dunbar, R. I. M. & Lamm, C. Being mimicked affects inhibitory mechanisms of imitation. *Acta Psychol. (Amst).* **209**, 103132 (2020).

7. Lang, P. J. International affective picture system (IAPS): Affective ratings of pictures and instruction manual. *Tech. Rep.* (2005).

8. Tarr, B., Launay, J. & Dunbar, R. I. M. Silent disco: dancing in synchrony leads to elevated pain thresholds and social closeness. *Evol. Hum. Behav.* **37**, 343–349 (2016).

9. Manninen, S. *et al.* Social laughter triggers endogenous opioid release in humans. *J. Neurosci.* **37**, 6125–6131 (2017).

10. Pearce, E., Launay, J. & Dunbar, R. I. M. The ice-breaker effect: singing mediates fast social bonding. *R. Soc. Open Sci.* **2**, (2015).

11. Tarr, B., Launay, J., Cohen, E. & Dunbar, R. Synchrony and exertion during dance independently raise pain threshold and encourage social bonding. *Biol. Lett.* **11**, 20150767 (2015).

12. van Doorn, J. *et al.* The JASP guidelines for conducting and reporting a Bayesian analysis. (2019).

13. de Jong, T. A bayesian approach to the correction for multiplicity. (2019).

14. Westfall, P. H., Johnson, W. O. & Utts, J. M. A Bayesian perspective on the Bonferroni adjustment. *Biometrika* **84**, 419–427 (1997).

15. Plank, I. S., Christiansen, L.-N., Kunas, S. L., Dziobek, I. & Bermpohl, F. Mothers need more information to recognise associated emotions in child facial expressions. *Cogn. Emot.* 1–14 (2022).

16. van den Bergh, D. *et al.* A Tutorial on Conducting and Interpreting a Bayesian ANOVA in JASP. *LAnnee Psychol.* **120**, 73–96 (2020).

1. The script was written for statistical tests with one comparison (e.g., Mann-Whitney-U, T-test or post-hoc test after an ANOVA). [↑](#footnote-ref-1)
